# The Use of Garlic Oil for Olfactory Enrichment Increases the Use of Ropes in Weaned Pigs

**DOI:** 10.3390/ani9040148

**Published:** 2019-04-05

**Authors:** Nicola Blackie, Megan de Sousa

**Affiliations:** 1 Pathobiology and Production Sciences, Animal Welfare Science and Ethics, Royal Veterinary College, Hawkshead Lane, Hatfield AL9 7TA, Hertfordshire, UK; 2 Centre for Equine and Animal Science, Writtle University College, Chelmsford CM1 3RR, Essex, UK; megandesousa@live.co.uk

**Keywords:** Pig, enrichment, welfare, tail biting, post-weaning, garlic oil, olfactory

## Abstract

**Simple Summary:**

Pigs are highly intelligent and can be prone to tail biting behavior if their environment is lacking in complexity, which is a serious welfare concern. For disease control and hygiene, pigs are often kept in semi-barren environments to separate them from their faeces; this may include slatted floors. Slatted pens are also cheaper to maintain with straw being expensive in many countries. To make the environment less barren, pigs are required by law to have environmental enrichment or “toys”. In this study, we designed a novel enrichment consisting of garlic-scented rope. We compared the pigs’ current enrichment with either unscented or garlic-scented ropes for two weeks after weaning. We found that the pigs spent more time interacting with the garlic rope and that more pigs used it. Pigs also showed a preference for the rope with 84% of focal pigs choosing the garlic over the control rope. When the garlic ropes were re-sprayed on day 8, we saw an increase in the number of pigs using the garlic rope and the time spent interacting with it. This indicates that this caught their interest again as interactions had decreased over time. This might be useful to encourage pigs to use this enrichment.

**Abstract:**

Pig producers are required to provide environmental enrichment to provide pigs the opportunity to perform investigative and manipulative behaviours (EU directive 2001/93/EC). Preventing enrichment from losing its novelty and decreasing the rate at which animals become habituated is important to maintain use of enrichment over time. A comparative study was formulated to identify whether weaner pigs housed in a semi-barren environment displayed a preference for olfactory enrichment compared to non-scented enrichment. Pigs (*n* = 146) were selected at 28 days old from two different batches (*n* = 76 and *n* = 70) and divided into pens. All pigs were given a control and a treatment (garlic scented) rope. Behavioural observations and rope interactions were assessed through direct observation. Throughout the entire study, the length of interaction with the garlic device was significantly higher (*p* < 0.02), indicating that there was a preference for olfactory enrichment compared to an odourless device. There was no significant occurrence of tail, ear, or flank biting in both batches. Weaner pigs showed a preference towards olfactory enrichment. Although habituation began to occur, this effect was mitigated by re-spraying the ropes, which resulted in increased interactions.

## 1. Introduction

Pigs within the EU must have access to environmental enrichment according to the EU directive 2001/93/EC. This enrichment must encourage pigs to undertake investigation and manipulation activities. The type of enrichment provided can influence how much the pigs use it. Those that stimulate pigs to forage and explore seem to keep pigs interested longer [1]. Pigs kept in an enriched environment post-weaning show more exploratory behaviours, less aggression, and appear to have a more positive affective state compared to those kept in barren environments [2,3]. Some enrichment types seem to be favoured in comparison to others, for example, if the enrichment has destructible, ingestible, or odorous properties, this may be more attractive to the pigs [1,4,5,6]. Enrichment devices that comprise of both olfactory and gustatory factors are highly important to pigs, due to their innate curiosity. Enrichment that supports rooting and foraging is also important as pigs kept in a semi-natural environment spend around half of their time foraging [7] and rooting has been shown to be a priority to pigs [6]. In a slatted environment, pigs may not have access to straw, which is noted to provide the best form of enrichment [8]. However, rope and other chewable substrates have been shown to be appropriate and that metal objects are not as suitable as an enrichment for pigs. Rope results in more interactions compared to chain, sawdust and shavings [9] and leads to more manipulation in finishing and early-weaned pigs compared to chain, pipe, and wood [10,11]. Flavoured ropes have been investigated to collect oral fluid from weaner and finishing pigs [12]; these ropes were soaked in sweet-citrus flavours (sucrose solution, apple juice, and pineapple juice). Sweet/citrus flavoured ropes did not increase oral fluid secretion and behaviour was not evaluated [12]. When plain (unflavoured) ropes were used to collect oral fluid, more that 80% of pigs chewed the ropes in the time that they were offered although this was influenced by housing, with straw based pigs taking longer to interact with the ropes and performing less chewing compared to slatted pigs [13]. This suggests that rope is a valuable enrichment item for pigs of all ages.

Pigs seem to be non-averse to garlic, with a study by Janz et al. [14] demonstrating that pigs offered garlic treated feed showed a preference through increased intake and liveweight gains. However, the levels of garlic inclusion might alter pigs’ perception with some studies showing improvements in feed intake [14,15] with the addition of garlic and others showing no effects [16]. Garlic has strong olfactory properties and as such, the aim of this experiment was to investigate the impact of soaking rope in garlic oil as a novel enrichment device for weaned pigs. To date, there have been limited studies looking at olfactory enrichment in pigs. 

## 2. Materials and Methods 

The study was conducted at Sturgeons Farm, a commercial breeder-finisher unit part of Writtle University College, between December 2015 and January 2016. The Writtle University College Ethics Committee approved this research (98337238-AW1). 

One hundred and fifty (Landrace X Large White) weaner pigs were recruited onto the study from two batches, the first containing 76 and the second 70. Four piglets had to be removed from the study due to ill health. Piglets were allocated to the pens at random with litters being mixed. All piglets were born in standard farrowing crates and management and nutrition of all sows and litters were equal; 3 rooms containing 4 crates were used for each batch. Piglets had access to a heated creep area and were offered creep feed prior to weaning (Ultima 1 Starter Feed). At birth, piglets were cross-fostered as required and received 1 mL of iron within the first 24 h. Tail and teeth reduction were both performed on all piglets as approved by the veterinary surgeon at the quarterly visit. The pigs were 4 weeks old at weaning and were housed in pens (25/pen, 6 pens over 2 replicates) according to weight. All pens measured 250 cm by 350 cm and had slatted plastic flooring. Each pen consisted of a single trough where feeding occurred at around 8:30 am and 5:00 pm to allow ad lib feeding, this coincided with the turning on and off of lights. Each trough allowed roughly four weaners to feed at one single time although this did not appear to cause aggression. The entire barn comprised of a fan assisted ventilation system, with a solitary fan in each pen leading to the exterior. Temperature in the barn rarely fluctuated; newly weaned pigs received a direct heat source to compensate for the lack of bedding. 

Pens had two close-knit chains of a 6 mm diameter and 1.8 m in length attached to a metal bracket with an equal distance of 45 cm apart. Attached to these chains were either a control (unscented) rope or treatment (garlic) rope, which were soaked in a concentrated garlic oil extract (Pegasus Health) mixed with purified water. This solution was used in a ratio of 30 mL (oil): 1 L (water) ropes were soaked individually for a 4-h period and dried overnight. All ropes were then attached 2 h before the pigs initially entered the pen. In the first batch of each pen, the control rope was attached to the right chain and the garlic flavoured to the left, then reversed in the second batch. The rope used in this study was natural cotton rope, measuring 50 cm in length with a diameter of 30 mm. Ropes were tied at the top, with the remainder of the fibres split into 3 individual ropes, thus allowing fraying at the base. The pigs had a standard piece of enrichment in addition, which was a porcichew attached to a chain. Garlic ropes were re-sprayed with a concentration of 10 mL:500 mL on day 8 of the trial and allowed to dry for 30 min before being returned to the pen. 

Behavioural observations were undertaken and consisted of focal observations. Ten focal pigs per batch were selected at random and marked with a stock marker to give individual identification; equal numbers of males and females were selected. Instantaneous focal sampling of behaviour was carried out at 2 min intervals for 1 h (*n* = 30 observations per hour observation period). The recorded behaviours were mutually exclusive and are detailed in the ethogram (Table 1) and all pigs were observed. Focal pigs were used to describe preferences for rope. 

Measurements of pigs interacting with ropes were collated by recording the length of interaction (start and stop time of interaction), number of individuals interacting, and the frequency of the interaction with each rope. One observer (MdS) spent an hour a day observing enrichment use, equating to 14 h of observations per pen of pigs over 2 weeks. This observation time varied to cover as much of the day as possible. To avoid an effect of the observer on pig behaviour, a habituation period of 15 to 20 min was allowed before each observation period. Rope interactions were collated from all pigs within each pen, however, animal preference was documented from the focal pigs in each pen. 

All data were tested for normality using the Pearson’s skewness test prior to analysis. From the scan sampling, the number of pigs interacting with the ropes within the hour of observation were added up for each day. These data were then analysed using a repeated measure analysis of variance with the repeated measure being the 14 days. For this test, the factors assessed were the effects of treatment, time, and the interaction between treatment and time. For the time spent interacting with the ropes, this was calculated from the focal pigs and this was also assessed using a repeated measures analysis of variance. The 30 observations per day were summed and these data were used. 

Time budgets and behaviours were statistically analysed using Kruskal-Wallis to identify the most performed behaviour alongside a Mann-Whitney *U* test as a post hoc test. All statistical analyses were completed through GenStat version 17 (VSN International Ltd., Hemel Hempstead, UK). 

## 3. Results

Differences were seen in the number and length of rope interactions when comparing the treatment (garlic treated rope) and control (untreated rope).

### 3.1. Activity Budget of Piglets

The most performed behaviour across both batches and all pens equalled ‘resting/sleeping’ (*p* < 0.001), indicating that weaner pigs spend the majority of their day asleep or resting (23.7% of observations). The next most prevalent behaviour of the focal pigs was interaction with the garlic rope, which made up 17.2% of their time (*p* < 0.002). Pigs also spent 14% of their time interacting with others and 4.3% of their time interacting with the control rope. Exploring the environment was undertaken in 12.1% of observations (*p* < 0.005). Upon re-spraying the garlic extract, an increase of the time spent interacting with the garlic rope was noted, with a 17.5% increase of time spent interacting was observed. There were no significant incidences of injurious behaviours performed throughout the study. 

### 3.2. Rope Interactions 

**Figure 1:**
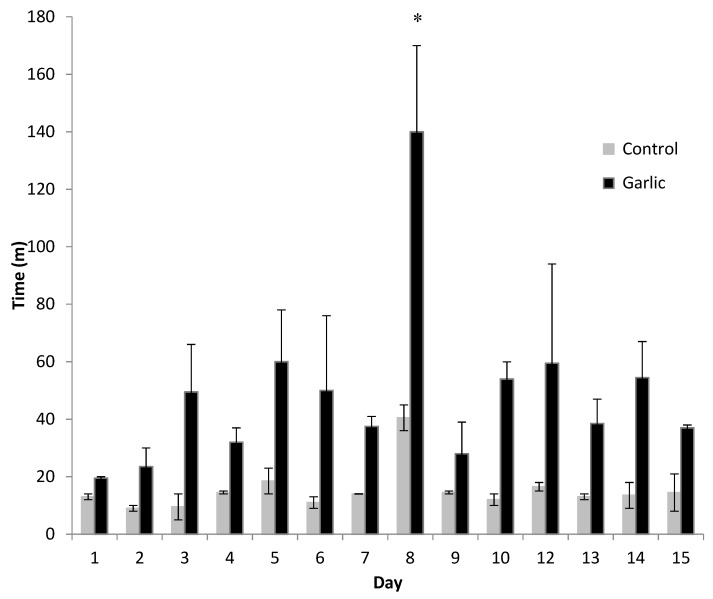
Length of time spent interacting with the control (untreated) rope compared with the garlic treated rope for each day over the study period. There was a significant effect of treatment (*p* = 0.017), but no effect of time (*p* = 0.159) or a treatment x time interaction (*p* = 0.307). The asterisk represents when the treatment rope was re-sprayed with garlic oil (day 8).

Soaking rope in garlic oil had an effect on the length of time individuals spent interacting with the rope compared to the control (unscented) enrichment device (*p <* 0.02, Figure 1). There was a rise in interaction with the garlic rope at the point of re-spray as seen in day 8 in Figure 1. At the point of re-spraying the garlic extract, the interaction levels increased compared to the day before re-spraying, a 73% increase in activity was recorded. 

**Figure 2:**
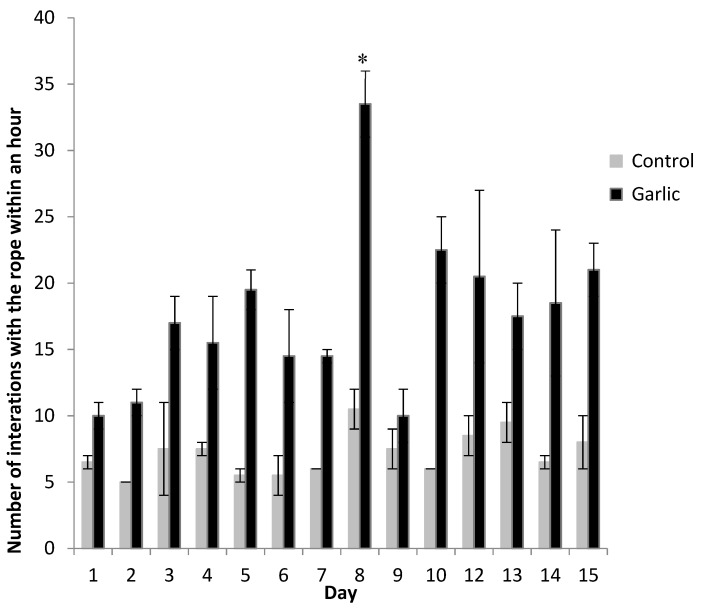
Number of interactions observed within an hour for the control (untreated) rope compared with the garlic treated rope each day over the study period. There was a significant effect of treatment (*p* = 0.004), but no effect of time (*p* = 0.143) or a treatment x time interaction (*p* = 0.242). The asterisk represents when the treatment rope was re-sprayed with garlic oil (day 8).

**Figure 3:**
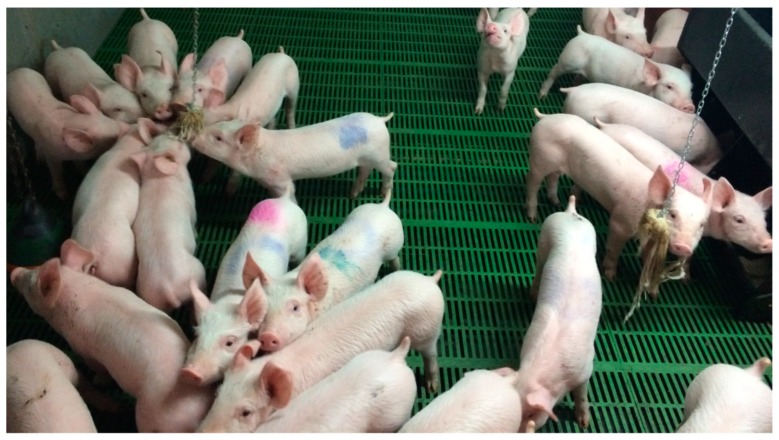
An example of pigs interacting with the garlic rope (left) or control rope (right). Focal pigs were marked with a spray marker to give individual identification.

With regards to the number of individual pigs interacting with the ropes, there were significantly more pigs interacting with the garlic treated rope (*p =* 0.004) within a one hour period of observation (Figure 2). The number of pigs interacting with the ropes showed a similar pattern to the amount of time spent interacting with the ropes. Again, an increase in the number of interactions was observed on the day on which the rope was re-sprayed (day 8). The number of pigs interacting increased by 57% in comparison to the previous day. Figure 3 shows the pigs using the rope enrichment; the garlic soaked rope is on the left and the control rope is on the right of the image.

Throughout the entire study, focal animals preferred the garlic rope by using the ropes for a greater amount of time. Individuals interacted with the garlic rope for an increased amount of time (>70% of individuals showed a garlic rope preference) compared to the control treatment. When focal pigs were seen interacting with the enrichment, 84% used the garlic rope and 16% used the control. 

## 4. Discussion

Given the popularity of the garlic scented rope expressed in both the time interacting with it and the number of pigs interacting with it, it would appear that it is an effective olfactory enrichment for pigs. Olfactory ques are used by pigs to recognize familiar conspecifics [17], locate the udder [18], and play an important part in feed palatability [19]. Piglets showed a preference towards olfactory enrichment and spent a lot of time of their day interacting with the device, distracting them from performing any injurious behaviours, which were not observed. Pigs in the present study were kept in a barren environment and spent proportionally more time interacting with the garlic rope and the environment than interacting with each other. Our study did not capture oral fluid from ropes like other studies [12,13], however, the use of garlic oil could be investigated to see if it improves fluid recovery. 

Habituation is described as the process of an object losing novelty and an animal no longer responding to an object’s presence [5]. Once an animal becomes habituated to a stimulus, the motivation to interact with the stimulus lessens. If the object is not initially attractive to the animal, interest will decrease quickly [5,20], thus leading to a potentially enriched environment becoming barren. Habituation occurs even when enrichment is rotated or changed [5,9], although the number of pigs interacting with each device began to decrease over time until the re-application of garlic oil; the interaction with garlic was always significantly higher than that of the control and other enrichment devices. Habituation may have taken place as is often seen with enrichment devices, however, once garlic ropes were re-sprayed, use of the devices increased by 35% in total. Interaction activity remained at an increased level post spraying for three days across all results at a level of 46%, supporting the theory of Van de Weerd et al. [1] of applying an odour to an enrichment device to reduce habituation. Length of interaction with the garlic rope increased following respraying, i.e., increased from 40 min/day to 140 min/day as well as the number of pigs using it. Interestingly, the control ropes were also manipulated more after the garlic ropes were refreshed. This may be facilitation behaviour or simply that not all the pigs could interact with the garlic rope at one time. Pigs who may want to interact with the garlic rope, but could not due to lack of space may have chosen to manipulate the control rope instead. 

This re-interest in the enrichment may be of particular importance in the event of a tail biting outbreak and it has been shown that extra enrichment can act as an intervention against tail biting [21]. The pigs in this study were docked to avoid outbreaks of tail biting and this is recognised as being in the pigs best interest [22]. We would like to repeat the study on undocked pigs to investigate the impact of our enrichment strategy on tail biting. 

The method of preference testing in this study was a simple choice test, rather than testing based on motivation and drive. This is mainly due to the nature of the intensive pig production system and the restrictions of altering the running of the unit. As motivation tests often rely on the animal experiencing some sort of cost to access the device [23], this is not ideal in a farming environment. Choice method testing proved successful in this study, as the aim was to identify whether pigs have a preference for olfactory enrichment over unscented rather than assessing how strong the motivation is to access the different enrichments. Although some of these methods do overlap, it is generally acknowledged that if an animal spends a greater quantity of time interacting with a certain device instead of another, then the animal prefers the primary stimulus [24]. It is argued that the resource the animal interacts with more is not necessarily preferred; however, this is unlikely in the present study due to a lack of fluctuations in the environment, social grouping, and fixtures and fittings [25]. An issue of laterality could possibly be raised in this study, as ropes scented or unscented were placed on the right or left. Farmer and Christison [26] stated that piglets and weaners did not show any laterality towards types of flooring as behaviours were equally performed regardless of whether specific flooring was located at the left or the right of the animal. While laterality research in pigs is limited, it is unlikely that laterality was present in this study and in fact, a preference for the garlic device was shown regardless of which side the treatments were offered.

## 5. Conclusions

The results from this study suggest that weaner pigs prefer olfactory enrichment to non-olfactory enrichment. From these results, it can be concluded that garlic is an attractive odour and flavour to juvenile pigs. When the ropes were re-sprayed, the novelty increased, suggesting the addition of scent reduces habituation to an enrichment device. Garlic scented ropes may be useful for distracting pigs when a tail-biting outbreak occurs.

## Figures and Tables

**Table 1:** Ethogram of behaviours recorded in weaned pigs.

Behaviour	Description
Standing	Pig stood on all four limbs stationary
Feeding/drinking	Pig consuming supplied feed from trough/Pig latched on drinker and swallowing water
Interacting with others	Pigs actively seeking other pen-mate and chasing, sniffing, belly nosing and other general interactions
Enrichment use	Interaction the ropes including sniffing, chewing, thrashing with rope in mouth or walking with rope
Exploring environment	Rooting or licking action around parameter of environment
Locomotion	Pig walking around the pen with no clear purpose other than to reach a different area/device
Aggression (fighting)	Individuals nosing, biting resulting in both parties rearing onto hind limbs and pushing each other until an individual has fallen
Tail/ear/flank, biting	Individual seeking out aforementioned appendage and biting
Resting/sleeping	Animal lying down with eyes open or closed
O.F.S. (out of sight)	Selected individual is not visible
